# Efficient Nebulization and Pulmonary Biodistribution of Polymeric Nanocarriers in an Acute Lung Injury Preclinical Model

**DOI:** 10.1002/smsc.202400066

**Published:** 2024-06-18

**Authors:** Anna Solé‐Porta, Aina Areny‐Balagueró, Marta Camprubí‐Rimblas, Elena Fernández Fernández, Andrew O’Sullivan, Rossella Giannoccari, Ronan MacLoughlin, Daniel Closa, Antonio Artigas, Anna Roig

**Affiliations:** ^1^ Institut de Ciència de Materials de Barcelona ICMAB‐CSIC Campus UAB 08193 Bellaterra Spain; ^2^ Critical Care Research Center Parc Taulí Hospital Universitari Institut d’Investigació i Innovació Parc Taulí (I3PT‐CERCA) Universitat Autònoma de Barcelona 08208 Sabadell Spain; ^3^ Esfera UAB‐CEI Universitat Autònoma de Barcelona 08193 Bellaterra Spain; ^4^ Centro de Investigaciones Biomédicas en Red de Enfermedades Respiratorias CIBERES‐Instituto De Salud Carlos III 28029 Madrid Spain; ^5^ Medical Affairs Aerogen Limited Galway Business Park H91 HE94 Galway Ireland; ^6^ R&D Science & Emerging Technologies Aerogen Ltd. IDA Business Park H91 HE94 Galway Ireland; ^7^ School of Pharmacy and Biomolecular Sciences Royal College of Surgeons Dublin D02 YN77 Ireland; ^8^ School of Pharmacy and Pharmaceutical Sciences Trinity College Dublin D02 PN40 Ireland; ^9^ Institut d’Investigacions Biomèdiques de Barcelona Consejo Superior de Investigaciones Científicas (IIBB‐CSIC) Barcelona 08036 Spain; ^10^ Servei de Medicina Intensiva Corporació Sanitària i Universitària Parc Taulí 08208 Sabadell Spain

**Keywords:** inhalation, lung, nanocarriers, poly(lactic‐co‐glycolic acid), vibrating mesh nebulizer

## Abstract

Acute respiratory distress syndrome (ARDS) is a clinical syndrome characterized by acute hypoxemic respiratory failure. Pneumonia and sepsis are the most common causes, turning ARDS into a critical public health problem. Despite recent advances in pharmacological strategies, clinical trials have not demonstrated a reduction in ARDS‐associated mortality. This is in part connected to the singularity of the pulmonary physiological barrier, which hampers drug delivery, specifically at distal areas. To this aim, the use of polymeric nanocarriers as a platform for the efficient delivery of therapeutics to the lungs by nebulization is introduced. Herein, poly(lactic‐co‐glycolic acid) (PLGA) nanocapsules (NCs) loaded with human serum albumin, as an inhalable nanotherapeutic are prepared. The production of stable NCs aerosols in the inhalable range is achieved using a commercial device, while the nanocarrier's physicochemical parameters are only minimally altered after nebulization. Importantly, in vivo studies with healthy and acute lung injury animals show that after inhalation, the NCs are homogeneously distributed throughout the lungs, arriving at the distal areas. The NCs are internalized by alveolar type II cells, avoiding macrophage‐mediated lung clearance. These features make the PLGA NCs excellent vehicles for noninvasive pulmonary delivery, facilitating a ready‐to‐be‐used nanomedicine.

## Graphical abstract

Poly(lactic‐co‐glycolic acid) nanocapsules (NCs) are investigated as inhalable nanotherapeutics. Stable aerosols can be produced using a commercial nebulizer without any significant alteration of the NCs. In vivo studies with healthy and acute lung injury animals show NCs homogeneously distributed throughout the lungs, arriving at even distal areas. NCs are internalized by alveolar type II cells, avoiding macrophage‐mediated lung clearance.

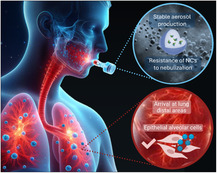

## 1 Introduction

Acute respiratory distress syndrome (ARDS) is a clinical syndrome of acute hypoxemic respiratory failure due to lung inflammation,^[^ ^1^ ^]^ which evolves from severe diffuse lung injury. It is associated with 10% of admissions to intensive care units and 45% of mortality in the critical category.^[^ ^2^ ^]^ Pneumonia and sepsis are the most common causes of this syndrome, and survivors often endure a reduced quality of life, turning ARDS into a public health problem and a tremendous burden on the healthcare systems worldwide.

Current treatments for ARDS include respiratory support and medication. Drug candidates for these conditions are continuously emerging, and over the last decade, the management of ARDS has made considerable progress in using mechanical ventilation as supportive therapy. In spite of supportive care of patients with ARDS, morbidity and mortality are still high. This is in part related to the uniqueness of the pulmonary physiological barrier with a relatively low lung drug delivery efficiency that severely reduces the efficacy of the administered drugs. Therefore, effective therapies focused on ARDS pathophysiology are needed. Currently, there is no specific pharmacological treatment that has yet proven benefits in clinical trials.^[^ ^3^, ^4^, ^5^ ^]^ Therapies targeting the alveolar‐capillary membrane, mucolytics, bronchodilators, immunomodulators, anticoagulants and fibrinolytics, aspirin, and other treatments are under investigation.^[^ ^6^, ^7^ ^]^ Some of the tested therapeutic agents are directed at the regeneration of the alveolar‐capillary membrane, some examples are: AP301/solnatide (a synthetic peptide that activates epithelial sodium channels), keratinocyte growth factor, vascular endothelial growth factor, anti‐inflammatories such as steroids, or even specific components from mesenchymal stem cells.

Nanomedicine‐based delivery systems, relying on nanocarriers specifically designed to improve pharmacokinetics and the therapeutic outcome of drugs, have already demonstrated high potential to treat several complex pathologies.^[^ ^8^, ^9^ ^]^ Likewise, a rational nanocarrier design and an adequate administration route will be necessary for the precise management of ARDS, as reviewed by Qiao et al.^[^ ^10^ ^]^ And even though many factors can affect the delivery and therapeutic efficiency of a treatment,^[^ ^11^ ^]^ two important features are pivotal in the context of ARDS: i) the need for the nanocarriers to accumulate with therapeutic dosage in all inflammatory lung sites; and ii) the benefit of using nanocarriers capable of loading multiple drugs to tackle pleiotropic pharmacological mechanisms synergistically.^[^ ^12^ ^]^ The ultimate aim is to decrease diffuse lung injury and promote the regeneration of the alveolar‐capillary membrane to reestablish lung function by considering individual patients’ needs and advocating for more personalized and precise treatments.

Intravenous administration of therapeutics is currently the preferred administration route in preclinical studies due to its rapid onset effect and easy dose control.^[^ ^13^ ^]^ However, the intravenous route still holds many drawbacks, including the harmful side effects associated with systemic drug distribution and the high accumulation of most nanomaterials in the liver, kidneys, and spleen, which may cause chronic injuries^[^ ^13^ ^]^ or immune‐mediated side effects such as infusion reactions to nanomedicines.^[^ ^14^ ^]^ Local, lung‐targeted administration can overcome the aforementioned drawbacks, and pulmonary delivery of nanomaterials is an advantageous strategy for lung disease treatment because of an increased accumulation in the target tissue compared to the intravenous route.^[^ ^15^, ^16^ ^]^ It could also enable local transfection in specific lung cells that are difficult to target through systemic delivery.^[^ ^17^ ^]^ Particularly, inhalation using appropriate devices—nebulizers, pressurized metered dose inhalers, nasal sprays, or dry powder inhalers—is a noninvasive approach favoring accumulation in the lungs.^[^ ^16^ ^]^ However, the efficacy of the nanoformulation delivery is affected by various factors, including mechanical clearance, mucociliary clearance, macrophage phagocytosis, and lymphatic transport in the respiratory tract.^[^ ^15^ ^]^ Furthermore, the complex inflammatory microenvironment associated with acutely injured lungs and impaired lung air–blood barrier may hinder drug entry and penetration into the injured tissue, affecting therapeutic efficacy.^[^ ^18^ ^]^ Thus, developing nanocarriers that can be nebulized and surpass these hurdles is a much sought‐after goal in the field.^[^ ^13^ ^]^

Nebulizers convert drug solutions or suspensions into small droplets that can be deposited in the lungs, offering the possibility to deliver relatively high doses over extended periods continuously,^[^ ^16^ ^]^ with minimal patient cooperation.^[^ ^19^ ^]^ The physicochemical properties of the aerosol formulation, such as viscosity, ionic strength, pH, and surface tension, affect the nebulization rates and the aerosol sizes,^[^ ^15^ ^]^ and can have the potential to damage the nanomaterials or even the active agent. Drug/device codevelopment is required for an optimal formulation and therapeutic delivery. Not all nebulizers are equivalent, providing a range of relative advantages and disadvantages. However, the development or selection of specific devices capable of achieving the appropriate aerosol performance metrics is a key enabling component of optimized treatment strategies.^[^ ^20^ ^]^

In this work, polymeric nanocapsules (NCs) are prepared by employing poly(lactic‐co‐glycolic acid) (PLGA), a synthetic biocompatible and biodegradable polymer approved by the Food and Drug Administration and the European Medicines Agency in a variety of drug delivery systems.^[^ ^21^, ^22^ ^]^ The spherical PLGA NCs of ≈250 nm diameter have an empty core suitable to accommodate multimodal drugs, including biological therapeutics, such as proteins or RNAs. This feature is purposely designed since most medical strategies proposed to treat ARDS aspire to have effectiveness across multiple pathways through the combination of several drugs. Human serum albumin (HSA) was selected as a model protein due to its medium size, availability, low cost, biocompatibility, and presence in some pharmaceutical products, such as Abraxane. Our previous studies showed that the encapsulation efficiency of HSA in these nanocarriers is around 50%, while the loading efficiency, defined as the mass of protein encapsulated versus the mass of the NCs, is ≈1%.^[^ ^23^ ^]^

Here, we report on the nebulization of PLGA NCs loaded with HSA (PLGA/HSA NCs) employing the most widely used vibrating mesh nebulizer (VMN), the Aerogen Solo, for inhaled drug therapy. This device is well described in the literature and has demonstrated compatibility with several other nanoscale formulations.^[^ ^24^, ^25^ ^]^ The first objective of this work is to assess whether the nanocarriers are suitable for nebulization in suspension, producing stable aerosols and withstanding the physical stress induced by the vibrating mesh. To this aim, the aerosol and NCs properties before and after the nebulization are thoroughly characterized. Second, the in vivo biodistribution and cellular uptake are evaluated in healthy and acute lung‐injured (ALI) rodents to determine the reach of the NCs in the lungs and to identify the principal NC‐uptaking cells.

## 2 Results and Discussion

The PLGA/HSA NCs used here as a model of a therapeutic nanocarrier for pulmonary administration via nebulization were synthesized by the double emulsion‐solvent evaporation route. Cyanine 5 anchored to PLGA‐NH_2_, modified from commercial PLGA, was used as a fluorescent probe for the in vivo experiments. First, the aerosol characteristics were investigated by laser diffraction, and then the main physicochemical parameters of the NCs pre‐ and postnebulization were analyzed by nanoparticle tracking analysis (NTA), dynamic light scattering (DLS), and scanning electron microscopy (SEM). Next, the in vivo biodistribution of fluorescent NCs in healthy and ALI animals was studied by fluorescence molecular imaging (FMI) and confocal microscopy, while the in vivo cellular uptake was assessed by flow cytometry.

### 2.1 Aerosol Characterization

Laser diffraction was used to characterize aerosol parameters: i) the aerosol size, defined by the volume median diameter; ii) the fine particle fraction, which corresponds to the percentage of droplets falling below 5 μm diameter; and iii) the flow rate, which indicates the speed of the aerosolized formulation. PLGA/HSA NCs were suspended in ultrapure water and 0.9% saline at three NC's concentrations (0.1, 1, and 5 mg mL^−1^).

**Figure 1:**
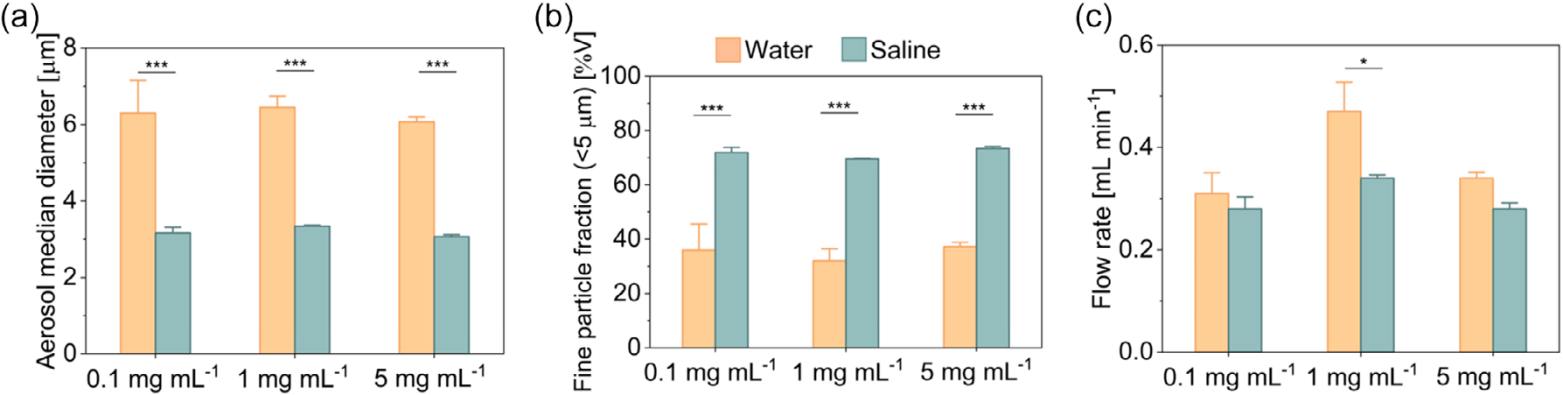
Aerosol characterization during the nebulization of PLGA/HSA NCs at different concentrations (0.1, 1, and 5 mg mL^−1^) in water and saline: a) aerosol droplet size (defined as volume median diameter); b) fine particle fraction (defined as the percentage of aerosol with a size below 5 μm); and c) flow rate in mL min^−1^. Data shown as the mean ± sem (*N* = 3); *p‐val < 0.05; ***p‐val < 0.001.

The droplet diameter of the aerosol produced with water as a liquid vehicle was larger (around 6 μm) for all tested concentrations when compared to saline, for which the median size was 3 μm (**Figure** **1**a). The latter falls within the favorable aerodynamic diameter range for an efficient lung deposition, typically between 1 and 5 μm,^[^ ^26^, ^27^, ^28^ ^]^ being 3 μm optimal for alveolar deposition.^[^ ^4^ ^]^ Related to this, the fine particle fraction was higher when using saline as a medium (≈70%) (Figure 1b). The data plotted in Figure 1c indicate that the flow rate of the nebulization of PLGA NCs suspensions was not affected by the NC concentration when tested with saline with a value around 0.3 mL min^−1^. Conversely, the flow rate tended to be more variable within the replicates and concentration when using water.

From the experimental data, we can infer that different formulations have a significant effect on aerosol performance. Saline would be preferred as a liquid carrier over water to produce PLGA NCs aerosols with the smallest droplet size and the highest fraction of droplets smaller than 5 microns. Moreover, the production of stable protein‐loaded NCs aerosols in the inhalable range was feasible, and they could be considered suitable candidates for efficient delivery of therapeutic agents via nebulization, favoring alveolar deposition.

### 2.2 Hydrodynamic Diameter and Concentration of the NCs

**Figure 2:**
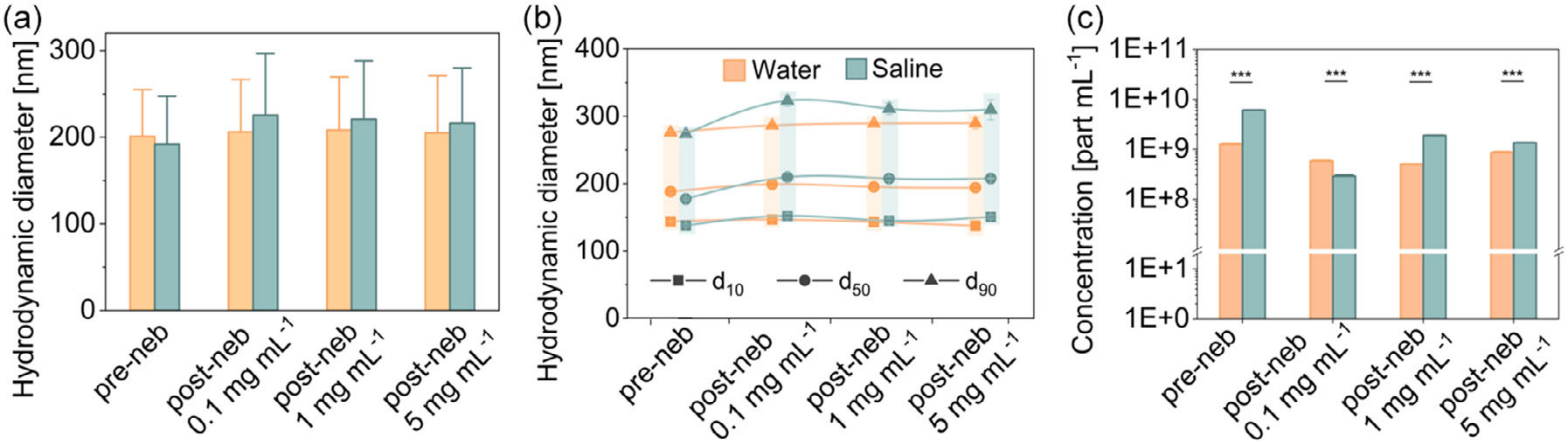
Hydrodynamic PLGA/HSA NCs diameter obtained by NTA before and after nebulization at different NC's concentrations (0.1, 1, and 5 mg mL^−1^) in water and saline; a) comparison of the hydrodynamic diameter mean values; b) 10th, 50th, and 90th percentiles; and c) concentration (error bars are present but hardly visible). Data shown as the mean ± sem (*N* = 3); ***p‐val < 0.001.

A thorough characterization of the NCs before and after nebulization was performed to analyze possible alterations of the physicochemical and morphological features of the carriers. First, the hydrodynamic size of the NCs was studied by NTA (intensity plots in Figure S1, Supporting Information). The mean hydrodynamic diameter of PLGA/HSA NCs pre‐ and postnebulization in water was between 200 and 210 nm, and it remained very stable regardless of the NCs concentration (**Figure** **2**a). In the case of saline medium, the size of the particles varied from 190 nm (prenebulization) to 230 nm (postnebulization). The results indicate that the nebulization process did not significantly alter the size of the nanocarriers. This was confirmed by DLS (Figure S2, Supporting Information). Note that the NCs particle size is similar to that of natural exosomes,^[^ ^29^ ^]^ extracellular vesicles released from cells for paracrine communication, indicating the potentiality of the NCs to reach the targeted cells.

Related to the mean hydrodynamic size, the 10th, 50th, and 90th percentiles are depicted in Figure 2b. The *d*_10_, for example, corresponds to the 10th percentile, that is, the diameter (in nm) below which 10% of the particles fall, i.e., 10% of all particles analyzed are below this size. In water, the 10th, 50th, and 90th percentiles remained constant before and after nebulization at different concentrations, but when saline was used, there was a slight increase postnebulization.

The NC's concentration pre‐ and postnebulization was also evaluated by NTA (Figure 2c). The NC loss after nebulization was ≈50% and ≈80% in water and saline, respectively. However, this decrease corresponds to a reduction in the number of NCs from 10^9^ NCs to ≈10^8^ NCs. Thus, the number of particles is still high. In parallel, the amount of unrecovered sample (≈12%) after the nebulization process was calculated by weighting the NCs mass of pre‐ and postnebulization. This should be considered when determining the optimal dose for in vivo experiments. When comparing the sample loss by weight with the NTA results, the percentages exhibit significant disparity. This observation is reasonable considering that NCs account for just 0.01% of the sample's weight (0.1 mg of NCs in 1 mL of water/saline), so the calculated sample loss by weight is primarily attributed to solvent loss.

### 2.3 NC Morphology

**Figure 3:**
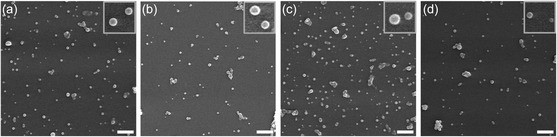
SEM images of PLGA/HSA NCs nebulized in water: a) prenebulization; postnebulization at b) 0.1 mg mL^−1^, c) 1 mg mL^−1^, and d) 5 mg mL^−1^. Scale bar: 2 μm.

The morphology of the NCs before and after nebulization at different concentrations was studied by SEM. In **Figure** **3**, only samples nebulized in water are shown to avoid image disturbances by the presence of sodium chloride crystals in the saline medium, in which NCs tend to accumulate (shown in Figure S3, Supporting Information). In all images, the particles remained spherical without major morphological changes and still monodisperse. Some small aggregates can be observed, which seem more abundant in the postnebulized NCs, but the difference is not significant.

Thus, the size, monodispersity, and morphology of PLGA/HSA NCs were only very lightly affected by nebulization, not causing major structural changes or impairing their main function, none while being dispersed in water or saline.

### 2.4 Lung Biodistribution of NCs In Vivo

**Figure 4:**
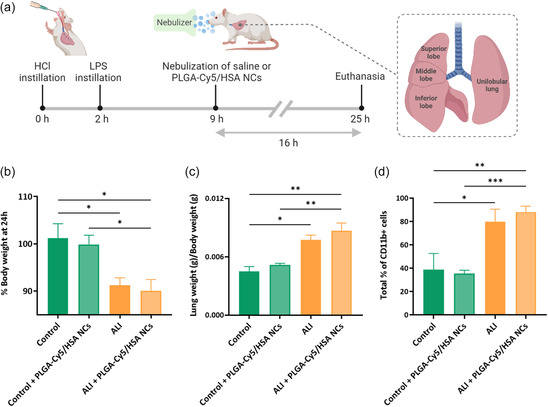
Experimental development and evaluation of the ALI animal model: a) in vivo experimental timeline and schematic illustration of the different lung lobes of a rat; b) animal body weight; c) ratio of lung/body weight; and d) infiltration of CD11b+ cells in lung tissue. Data shown as the mean ± sem (*n* = 2–6); *p‐val < 0.05; **p‐val < 0.001; ***p‐val < 0.001.

Once the compatibility of the PLGA NCs and VMN was demonstrated, we next assessed the NC's in vivo biodistribution in healthy and in an ALI Sprague–Dawley rat model after fluorescent PLGA‐Cy5/HSA NCs were administered by inhalation using the same nebulizer as for the in vitro studies (experimental timeline shown in **Figure** **4**a). The animals that received HCl and lipopolysaccharide (LPS) (ALI model) presented a more pronounced body weight loss after 24 h than healthy animals (Figure 4b). In addition, injured animals showed a significantly higher lung weight/body weight ratio (Figure 4c) and infiltrated immune cells (CD11b+ cells) in lung tissue (Figure 4d), indicating enhanced permeability of the alveolar‐capillary membrane and lung injury.

**Figure 5:**
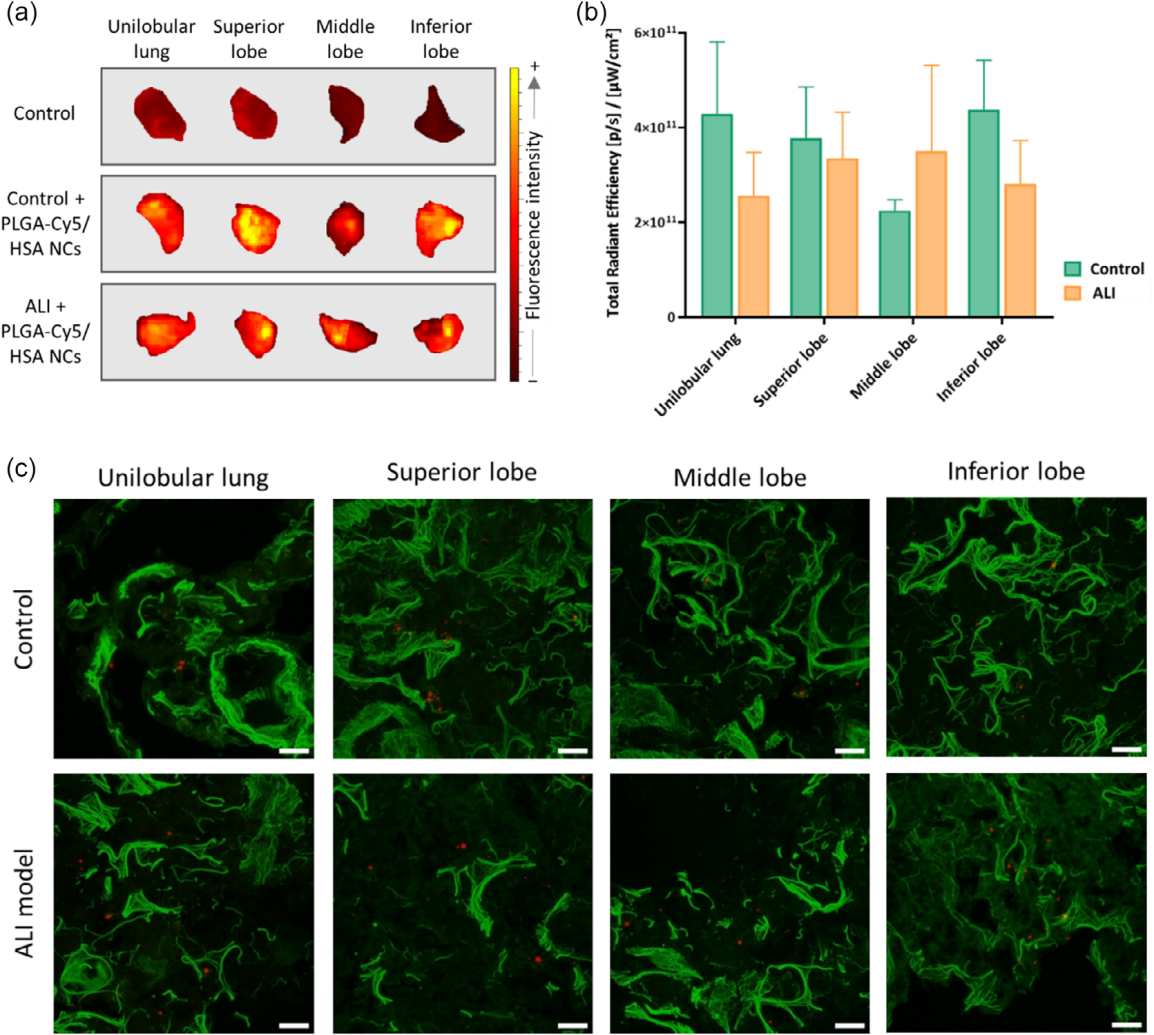
In vivo biodistribution of PLGA‐Cy5/HSA NCs in control (healthy) and ALI animals: a) FMI views of the lungs of a control animal, a healthy animal nebulized with NCs, and an ALI animal nebulized with NCs; b) corrected TRE on unilobular lung, superior, middle, and inferior lobes regions of interest (ROIs) of control (healthy) and ALI animals nebulized with NCs; and c) *Z*‐stacking analysis of lung (unilobular lung, superior, middle, and inferior lobes) tissue slices of animals nebulized with NCs. Scale bar: 20 μm. Green: membranes stained with Cell Mask; red: NCs (Cy5); 60× magnification; average zoom = 1×. Data is shown as the mean ± sem (*n* = 6).

FMI confirmed the presence of PLGA‐Cy5/HSA NCs in the lungs of healthy and ALI model animals after 16 h of nebulization by increased fluorescence intensity (**Figure** **5**a) compared to the lungs of animals that did not receive NCs. The total radiant efficiency (TRE) values obtained by FMI were equal and homogeneously spread in both lungs (uni‐ and multilobular) (Figure 5b). Although the TRE in the lungs of ALI animals was not as high as in healthy animals, differences in the retention of the NCs in lung tissue between healthy and injured animals were not significant (Figure 5b). Slight differences in the deposition of NCs in healthy and injured animals might be influenced by several anatomical, physiological, and immunological barriers that affect the delivery efficacy of inhaled biologics,^[^ ^30^ ^]^ such as the alveolar‐capillary membrane disruption and the infiltration of immune cells.^[^ ^31^ ^]^

These data were reinforced by confocal microscopy, in which we observed NCs (red spots) in all the lung lobes (Figure 5c), even in those with impaired alveolar architecture. In addition, the biodistribution of the NCs seemed to be homogeneous throughout the lung tissue since Cy5 fluorescence was present in the different lobes of healthy and ALI animals. Confocal microscopy images of control animals (nebulized with saline, no NCs) are shown in Figure S4, Supporting Information. The ALI model induced by HCl/LPS causes a diffuse injury at the different lung lobes. Thus, the arrival of NCs in all of them indicates the possibility of treating all the injured parts of the lung by nebulizing suitable nanocarriers.

Note that here, animals were spontaneously breathing while being nebulized. Although loss of NCs is expected, a successful arrival and accumulation of NCs is demonstrated in the distal areas of the lung. Besides, when nebulizing NCs to patients receiving mechanical ventilation, the loss of the NCs will be much lower than by the spontaneous breathing of rats.^[^ ^4^ ^]^

### 2.5 Cellular Uptake of the NCs In Vivo

Here, we focused on the ability of ATII and myeloid cells to internalize the PLGA/HSA NCs since they are the first line of host defense and innate immunity, which is essential for the maintenance of lung homeostasis.^[^ ^32^ ^]^ Moreover, ATII cells are the progenitor cells of the alveolar epithelium, proliferating and differentiating into ATI cells, being the major promoters of the reepithelization of the alveolar membrane for lung function reestablishment.^[^ ^33^ ^]^

**Figure 6:**
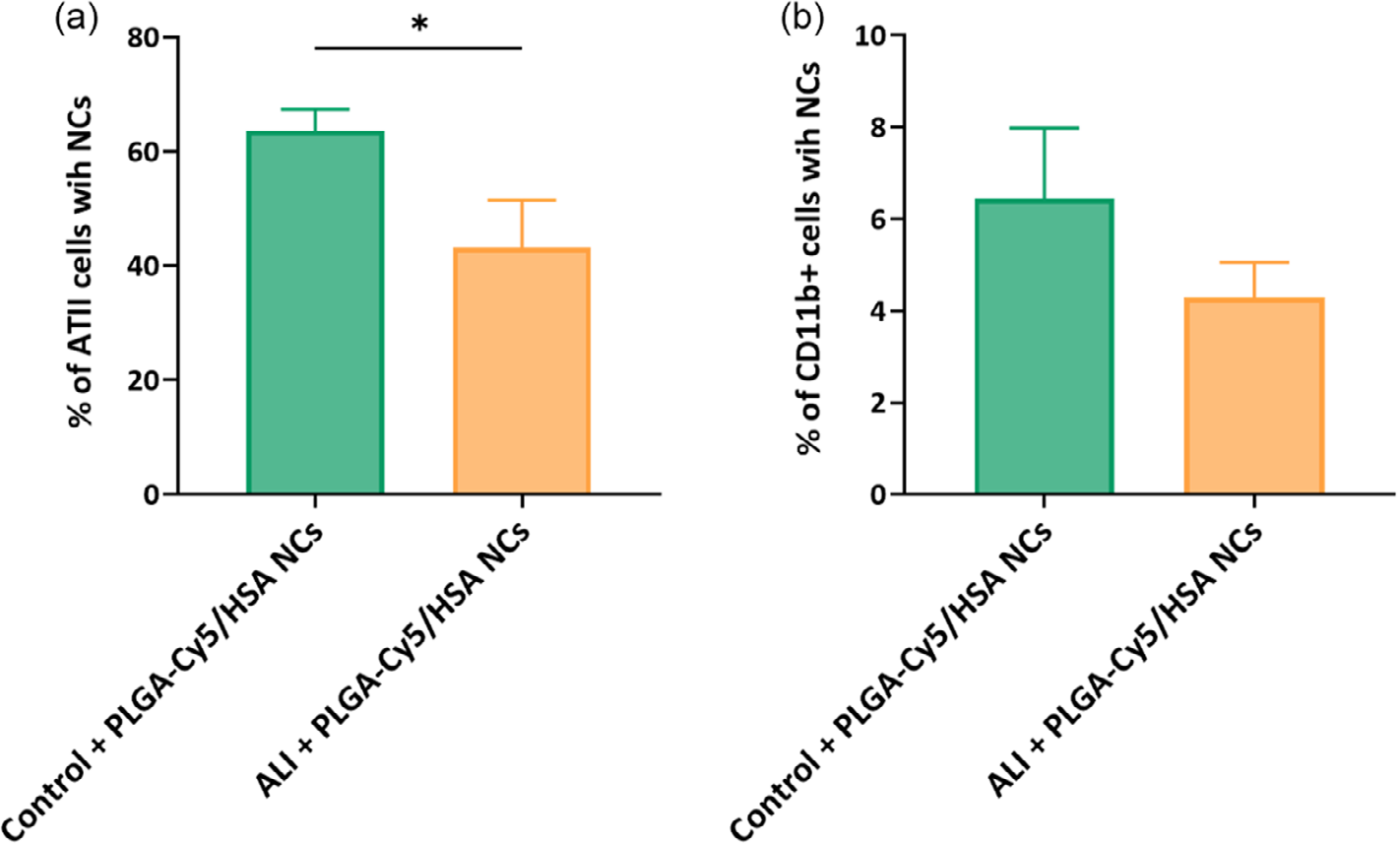
In vivo cellular uptake of PLGA‐Cy5/HSA NCs in control (healthy) and ALI animals: percentage of a) ATII cells and b) CD11b+ cells from lung tissue homogenates that were positive for Cy5 fluorescence. Healthy (green) and ALI animals (orange) were nebulized with PLGA‐Cy5/HSA NCs and uni‐ and multilobular lungs were analyzed. Data shown as the mean ± sem (*n* = 6); *p‐val < 0.05.

By flow cytometry analysis, ATII cells from healthy animals presented a significantly higher percentage of internalization of NCs compared to injured animals, showing 64% and 43% retention, respectively (**Figure** **6**a).

A challenge for cellular uptake of NCs by ATII might be the impaired mucociliary clearance and pulmonary surfactant (PS),^[^ ^34^ ^]^ both main defenses of the respiratory system.^[^ ^35^ ^]^ In addition, PS has been investigated as a carrier to promote the transportation of drugs in the airways thanks to its main proteins, such as surfactant protein A and C, SPA and SPC,^[^ ^36^ ^]^ which are decreased in ALI models.^[^ ^37^ ^]^ Altogether, this could limit NCs absorption and prevent them from close contact with lung epithelium.

Regarding the myeloid cells (CD11b+), which include macrophages, monocytes, and neutrophils, their average percentage of NCs’ internalization was higher in healthy animals (6.4%) than in those with an altered alveolar structure (4.2%) (Figure 6b), although injured animals presented a higher percentage of CD11b+ cells (Figure 4d) due to filtered immune cells.

Alveolar macrophages have a significant role in the clearance of inhaled particles from the alveolar region of the lungs, where there is an absence of significant ciliated epithelia and mucus.^[^ ^38^ ^]^ Lung inflammation impairs alveolar macrophages’ mobility and decreases the macrophage‐mediated lung clearance of the PLGA NCs, likely again as a result of impaired mucus viscosity^[^ ^39^ ^]^ and a reduction of SP, which could explain the reduced percentage of CD11b+ cells that captured NCs in injured animals.

Although it has been reported many times that alveolar macrophages should be one of the targets for pulmonary administered therapies due to their key role in triggering inflammatory processes in many pulmonary diseases,^[^ ^40^ ^]^ it has also been demonstrated that inhaled nanomedicines have a distinct benefit in pulmonary distribution by preventing macrophage absorption which is critical in reducing drug loss and maximizing drug retention.^[^ ^41^ ^]^ In addition, if the rate of inhaled particle deposition exceeds the rate of clearance, it could overload the macrophages with adverse inflammatory consequences.^[^ ^42^ ^]^ In our previous study, rats were intratracheally instilled with PLGA NCs and we observed that ≈98% of monocytes phagocytosed NCs after 24 h.^[^ ^43^ ^]^ In the present work, we observed a much lower percentage of NCs uptake by CD11b+ cells, which suggests that aerosol‐mediated delivery reduces the macrophage‐mediated lung clearance of PLGA NCs, promoting major tissue retention.

In the early phase of ARDS, neutrophils are recruited to the injured lung to promote pathogen clearance and avoid the spread of the injury.^[^ ^44^ ^]^ This, together with the fact that impaired macrophages cannot engulf neutrophil extracellular traps or apoptotic cells,^[^ ^45^ ^]^ explains the increased percentage of myeloid cells in injured animals. However, the percentage of myeloid cells that captured NCs was quite low, probably due to the inflammatory lung environment impairing inflammatory cell functions.

Overall, these results support the successful alveolar deposition of the NCs in the different lobules of the lung and their capture by ATII cells, the principal actors of lung immunity and regeneration. Myeloid cells, mainly involved in inflammation, also internalize the NCs but to a lesser extent, suggesting that nebulization is a more suitable administration method to enhance NCs’ tissue retention and avoid macrophage‐mediated clearance, which can affect the efficacy of delivered drugs. However, the interaction between ATII and immune cells is crucial in the lung environment to ensure the maintenance of lung homeostasis in normal and pathological conditions.^[^ ^32^ ^]^ Therefore, the fact that our nanocarrier is efficiently uptaken by ATII cells guarantees that the effect caused to them will also be reflected in the activity of immune cells. Nevertheless, further modifications of the nanocarrier with specific ligands can be included to direct them to specific target cells. In this line, the therapeutical agents encapsulated in the NCs can influence a combination of pathways and mechanisms involved in ARDS by acting on different cell types.

## 3 Conclusion

In the present work, we propose biocompatible and biodegradable PLGA nanocarriers, similar in size to extracellular vesicles used in paracrine communication, as promising nanoplatforms for noninvasive administration of therapeutic agents to the lungs by inhalation as a strategy to treat ARDS. The production of stable protein‐loaded NCs aerosols in the inhalable range was proven feasible, while the main physicochemical features of the NCs were very lightly affected by nebulization, not causing major structural changes nor impairing their main function. Regarding the biodistribution of the NCs in vivo, the presence of NCs in the lungs of healthy and ALI animals after nebulization was confirmed by FMI. This was supported by confocal microscopy images, which showed NCs in the unilobular lung and the superior, middle, and inferior lobes of the multilobular lung, even in ALI animals.

The cellular uptake of the NCs by ATII cells after nebulization was significantly higher in healthy animals compared to injured rats, which could be due to an increased permeability of the alveolar‐capillary membrane in the ALI model. In addition, nebulization reduced the macrophage‐mediated lung clearance of our PLGA NCs, promoting NCs’ tissue retention. Overall, PLGA NCs are excellent vehicles for noninvasive pulmonary delivery due to their capacity to withstand nebulization with excellent properties of the produced aerosols, the ability to accumulate in all the lung lobes, and the capacity to target ATII cells, the main actors of lung immunity and regeneration. In this work, a model protein was used to test the carrier, but further modifications of the formulation accommodating therapeutic agents inside the NCs are feasible to develop personalized treatments that can influence diverse pathways involved in ARDS.

ARDS has a complex pathophysiology that includes crosslinked mechanisms, leading to patient heterogeneity and hindering the possibility of using the same protocol for all patients, which underpins the need for personalized treatment. Nanomedicine can enable this approach as our NCs can be loaded with several drugs targeting different pathways. Moreover, PLGA NCs offer the possibility to be available at any time at the bedside, thanks to their stability after cryo‐storage, facilitating ready‐to‐be‐used medicinal products and their controlled delivery to the desired target organs and cells.

## 4 Experimental Section

4.1

4.1.1

##### Materials

PLGA (ResomerRG502H, acid terminated, lactide:glycolide 50:50, MW = 7–17 kDa), polyvinyl alcohol (87–90% hydrolyzed, MW = 3–7 kDa), triethylamine (≥99.5%), and HSA (lyophilized powder, essentially globulin free, ≥99%, agarose gel electrophoresis) were obtained from Sigma‐Aldrich. D(+)‐trehalose 2‐hydrate was purchased from AppliChem Panreac. Cyanine5‐NHS ester (Cy5, 95%) was obtained from Lumiprobe. Ultrapure water was produced with Milli‐Q Advantage A10 equipment from Millipore. All other chemicals and reagents were of the highest purity grade commercially available.

##### Synthesis of Fluorescent PLGA (PLGA‐Cy5)

First, an amino group was introduced as an end group in PLGA to form amino‐terminated PLGA (PLGA‐NH_2_), following previously established protocols.^[^ ^23^, ^43^ ^]^ Then, PLGA‐NH_2_ (100 mg, 0.008 mmol) and Cy5 (7 mg, 0.01 mmol) were dissolved in 3 mL of acetone. As a catalyst, a solution of trimethylamine (2.5 mg, 0.025 mmol) in 0.6 mL of acetone was added dropwise to the mixture, which was left under magnetic stirring for 6 h at room temperature (rt) in dark conditions. The resultant solution was slowly added to a 10‐fold excess amount of ethanol to force the precipitation of PLGA‐Cy5. The solid was settled for 10 min and then isolated by centrifugation at 9000 rpm (9435 g) for 10 min at rt. The obtained PLGA‐Cy5 pellets were dried under vacuum overnight in dark conditions.

##### Synthesis of HSA‐Loaded PLGA NCs

PLGA NCs loaded with HSA (PLGA/HSA NCs) were prepared by the double emulsion‐solvent evaporation method,^[^ ^23^, ^43^, ^46^, ^47^ ^]^ in which an HSA solution (50 μL, 20 mg mL^−1^) as the inner aqueous phase (*w*_1_), was added to 500 μL of dichloromethane (DCM), which is the organic phase (o). The latter contained 50 mg of different proportions of PLGA and PLGA‐Cy5. To obtain fluorescent NCs, a formulation was carried out by mixing PLGA‐Cy5 with as‐received PLGA at a 1:1 ratio, while for nonfluorescent NCs, only as‐received PLGA was used. A first emulsification was done by sonication at 200 W for 28 s to form a water‐in‐oil emulsion (*w*_1_/o). Then, an aqueous polyvinyl alcohol solution (2 mL, 20 mg mL^−1^), which is the external aqueous phase (*w*_2_) and acts as a stabilizer, was added and a water‐in‐oil‐in‐water emulsion (*w*_1_/o/*w*_2_) was formed by sonicating again at 200 W for 28 s. The temperature during the whole emulsion process was kept at 4 °C using an ice bath.

The resulting double emulsion was poured into 50 mL of ultrapure water (MilliQ) and mechanically stirred at 280 rpm at rt for 2 h. Then, the NCs were purified by centrifugation in ultrapure water at 4 °C. Centrifugation was performed at 900 rpm (943 g) for 15 min, followed by two centrifugations at 9000 rpm (9435 g) for 15 min. The NCs were finally redispersed in 6 mL of a 2 mg mL^−1^ trehalose aqueous solution, acting as a cryoprotectant. The solution was frozen at −80 °C for a posterior lyophilization process (LyoMicron −85 °C, Coolvacuum) for 72 h to preserve the NCs from hydrolytic degradation. The as‐obtained NCs powder was stored at −80 °C until its use. The stability of the lyophilized PLGA NCs is ensured during 2 months of cryo‐storage, as shown in a previous study.^[^ ^43^ ^]^

##### In Vitro Nebulization

**Figure 7:**
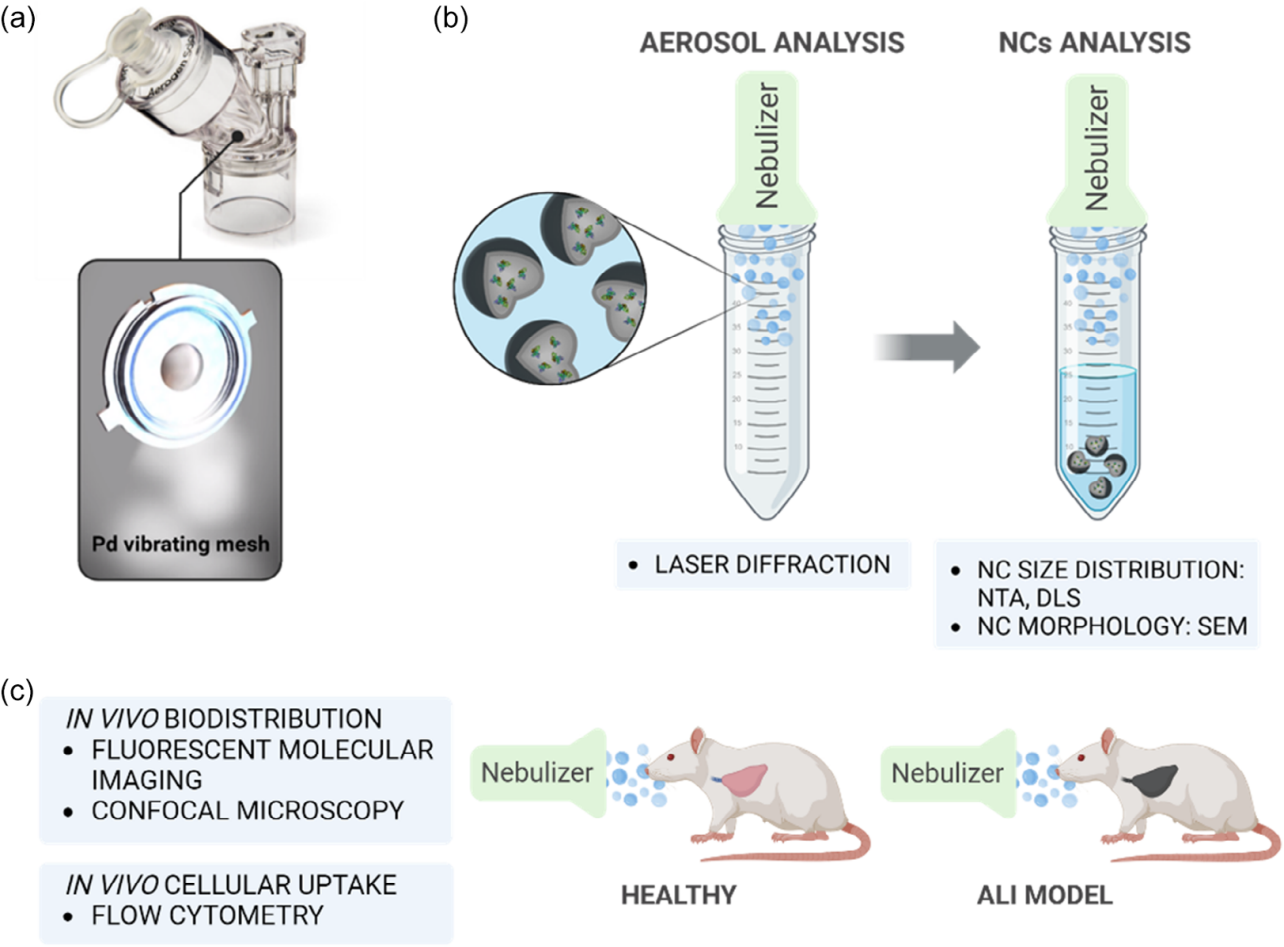
Scheme of the experimental work: a) commercial nebulizer (Aerogen Solo); b) in vitro nebulization procedure and analytic techniques used for aerosol and nanocarriers characterization; and c) in vivo experimental procedure and techniques used (animals were confined in restraint tubes and spontaneously breathing).

HSA‐loaded PLGA NCs were reconstituted in ultrapure water or 0.9% saline solution (0.9% NaCl in ultrapure water) at rt. Nebulization was conducted with a VMN (Aerogen Solo Aerogen Ltd with Aerogen ProX controller, Galway, Ireland), as shown in **Figure** **7**a. For this, suspensions (1 mL) at different concentrations of NCs (0.1, 1, and 5 mg mL^−1^) were nebulized into a 50 mL centrifuge tube affixed to the nebulizer and collected after nebulization was completed (Figure 7b). During and after the nebulization process, several techniques were used to characterize the aerosol and the NCs.

##### Aerosol Characterization

The aerosol droplet size produced by the VMN was characterized using laser diffraction (Spraytec, Malvern Instruments, Malvern, UK) as previously described.^[^ ^48^ ^]^ Briefly, the VMN was attached to the laser diffraction equipment, and a continuous vacuum flow rate of 5 L min^−1^ was applied to ensure a single pass of aerosol droplet through the laser and, thus, greater accuracy of results. The droplet size, defined in terms of the volumetric mean diameter (μm) and aerosol output flow (mL min^−1^), was recorded (3 replicates). Several combinations of VMN and NC formulation were characterized for droplet size and output rate, testing ultrapure water and 0.9% saline as media.

##### NTA

NTA was performed with a Nanosight NS300 (Malvern Instruments, Malvern, UK) equipped with a 488 nm laser to obtain the hydrodynamic diameter (*d*_H_) and the concentration of NCs before and after the nebulization. All suspensions were diluted in ultrapure water or saline to a 0.1 mg mL^−1^ concentration and loaded into the sample chamber. The scattered light was visualized by a 20× magnification microscope equipped with a sCMOS (scientific Complementary Metal Oxide Semiconductor) camera, which allowed us to record real‐time videos. Measurements were performed in triplicate. Automatic data analysis was performed on recorded data using the NTA 3.4 software.

##### DLS

The hydrodynamic diameter of the NCs was also measured by DLS, while zeta (ζ) potential measurements were performed by electrophoretic light scattering. Both magnitudes were obtained with a Zetasizer Nano ZS (Malvern Instruments, Malvern, UK), performing 3 replicates. The samples before and after nebulization were diluted to a NC's concentration of 0.1 mg mL^−1^ using the corresponding medium (ultrapure water or 0.9% saline).

##### SEM

A field‐emitting scanning electron microscope (SEM, FEI Quanta 200 FEG) was used to study the morphology of NCs. The samples before and after nebulization were diluted to a NC's concentration of 0.1 mg mL^−1^ using ultrapure water or 0.9% saline, and 10 μL of the slightly turbid suspension were placed onto a small piece of a silicon wafer stuck on the top of a carbon layer. The sample was dried at rt overnight and then sputtered with 60/40 Au/Pd (20 mA for 2 min to have 10 nm deposition) with an Emitech K550 instrument (Quorum Technologies Ltd.). Secondary electron images were taken using a working distance of 8 mm, a large field detector, an acceleration voltage of 15 kV, and a pressure of 60 Pa.

##### In Vivo Nebulization of NCs in a Preclinical Animal Model of ALI

Male (*n* = 9) and female (*n* = 9) Sprague–Dawley rats (Charles River, Écully, France), weighing 200–225 g, were used following the European Commission Directive 86/609/EEC and the Spanish guidelines for experimental animals. The experimental protocol was approved by the Animal Research Ethics Committee of the Autonomous University of Barcelona (UAB) and the Animal Experimentation Committee of the Generalitat de Catalunya, with the animal studies approval number 9455. Animals were kept under controlled environmental conditions; enrichment material was introduced inside the cages and animals were continuously supervised throughout the experiment. Food and water were available ad libitum.

ALI was induced as in our previous study.^[^ ^31^ ^]^ Animals received an intratracheal administration of 300 μL of HCl (0.1 M at pH = 1.4), and 2 h later, intratracheal instillation of the endotoxin LPS (*Escherichia coli* 055:B5, Sigma, St. Louis, MO, USA, 30 μg g^−1^ of body weight) dissolved in 300 μL of saline under sevoflurane anesthesia. Healthy animals did not receive any instillation, as we aimed to maintain their alveolar structure to the maximum to compare the biodistribution and cellular uptake of NCs in a normal and an affected lung architecture. After 9 h, HSA‐loaded fluorescent PLGA‐Cy5 NCs (PLGA‐Cy5/HSA), at a concentration of 7 mg mL^−1^, diluted in saline to a total volume of 700 μL were administered by inhalation using an Aerogen Solo nebulizer to healthy and injured animals, at a rate of 0.3 mL min^−1^. This system was connected to restraint tubes where animals were confined, allowing direct exposure of the nebulized agents to the rats, which were spontaneously breathing. Control animals with no fluorescence were nebulized with saline. The animals were anesthetized intraperitoneally with ketamine (90 mg kg^−1^)/xylazine (10 mg kg^−1^) (3:1) and were euthanatized by exsanguination of the abdominal aorta 16 h after.

The animal body weights and lung weights were recorded at the beginning and the end of the experiment to determine lung injury.

##### Lung Biodistribution of Nanocapsules In Vivo

NCs’ in vivo biodistribution was analyzed by ex vivo and histological studies. First, FMI was performed to characterize the fluorescence signal intensity of the NCs in the lungs using an IVIS Lumina Ex Vivo Imaging System (Perkin Elmer) working at *λ*_ex_/*λ*_em_ 640/732 nm, respectively. To quantify the TRE, ROIs were manually drawn and the TRE, expressed in μW cm^−^^2^, was measured using the Living Image software. The TRE values obtained from the lungs with no NCs were considered background noise and were used for correction. Each lobule was corrected by their respective controls (healthy or injured lungs).

In addition, histological analyses were done to gain a deeper insight into the NCs’ biodistribution. The unilobular and multilobular lungs (each lobule separately) were embedded in Tissue‐TekTMCRYO‐OCT (Science Services) and processed in 14 μm sections with a cryotome. Plasma membranes of the histological slices were stained with a green Cell Mask (Thermo Fisher Scientific) and mounted with Fluoromount Aqueous Mounting Medium (Sigma, St. Louis, MO, USA). Four slices from each sample were analyzed using a confocal microscope (Inverted Microscope Leica DMI 4000B), and Z‐stacking was recorded at a 1 μm interplanar distance. Green and red fluorescence were obtained using 488 and 635 nm laser excitation, respectively, 60× magnification, and an average zoom = 1. Images were processed using the software platform of LAS X Life Science (Leica Microsystems, Wetzlar, Germany) combined with ImageJ (X64, v. 2.1.4) software.

##### Cellular Uptake of NCs In Vivo

In vivo cellular uptake of NCs was studied by flow cytometry. Unilobular and multilobular lungs were removed and perfused with a constant flow of 25 mL of 0.25% trypsin solution for 15 min. After that, fetal bovine serum was added to stop trypsin activity, and then the lungs were chopped into 1–2 mm^3^ cubes, treated with DNase I (75 U mL^−1^), and filtered through nylon meshes (pore size 40–100 μm). Then, 2 mL of the resulting cell suspension was centrifuged and treated with ammonium chloride potassium (ACK) buffer to lysate the erythrocytes. After washing and Fc blocking with CD16/CD32 antibody, the cells were stained with the antibody mix at 4 °C in the dark (**Table** **1**). After 30 min of incubation, cells were washed and measured using a Fortessa4L cytometer for different cell leukocyte subsets’ counts, measurements, and classification. Data were analyzed using FlowJo.

**Table 1:** List of conjugated antibodies used in the flow cytometry analysis.

Antibody	Fluorochrome	Source	Reference	Dilution
*CD45*	PE‐Cy7	BioLegend	202 214	1:200
*CD11b*	Pacific Blue	BioLegend	201 817	1:200
*EpCAM*	Alexa Fluor 594	Santa Cruz Biotechnology	202 214	1:200
*APK*	Alexa Fluor 546	Santa Cruz Biotechnology	sc‐271 431	1:50

Cell subset populations were gated as follows selecting singlets; total myeloid cells: CD45+ and CD11b+; epithelial ATII: CD45+, CD11b‐, Ep‐CAM+, and APK+ (alkaline phosphatase). Myeloid cells and ATII cells that were positive for Cy5 fluorescence were counted to obtain the percentage of each population that captured NCs.

##### Statistical Analysis

All the data were analyzed using Origin 2023b and GraphPad Prism 7 software and expressed as the mean ± standard error of the mean (sem). One‐way ANOVA with Newman–Keuls multiple‐comparison test was applied to compare more than two groups and two‐way ANOVA followed by Bonferroni's multiple comparison test was used to analyze data with more than one variable. All statistical tests conducted are two‐sided and *p* < 0.05 is considered significant.

## Conflict of Interest

The authors declare no conflict of interest.

## Supporting information

Supplementary Material

## Data Availability

The data that support the findings of this study are available from the corresponding author upon reasonable request.
