# Supplementary material for: Efficient Nebulization and Pulmonary Biodistribution of Polymeric Nanocarriers in an Acute Lung Injury Preclinical Model
Source: Small Sci. 2024 Jun 18;4(9):2400066. doi: 10.1002/smsc.202400066 (PMC11935039; doi:10.1002/smsc.202400066)

Supporting Information

Efficient Nebulization and Pulmonary Biodistribution of Polymeric Nanocarriers in an Acute Lung Injury Preclinical Model

Anna Solé-Porta[1], Aina Areny-Balagueró[2,3,4], Marta Camprubí-Rimblas[2,3,4], Elena Fernández Fernández[5], Andrew O'Sullivan[6], Rossella Giannoccari[6], Ronan MacLoughlin[6,7,8], Daniel Closa[9], Antonio Artigas[2,3,4,10], Anna Roig[1,]*

[1] Institut de Ciència de Materials de Barcelona, ICMAB-CSIC, Campus UAB, 08193 Bellaterra, Spain

[2] Critical Care Research Center, Parc Taulí Hospital Universitari, Institut d'Investigació i Innovació Parc Taulí (I3PT-CERCA), Universitat Autònoma de Barcelona, 08208 Sabadell, Spain

[3] Universitat Autònoma de Barcelona, 08193 Bellaterra, Spain

[4] Centro de Investigaciones Biomédicas en Red de Enfermedades Respiratorias, CIBERES-Instituto De Salud Carlos III, 28029 Madrid, Spain

[5] Medical Affairs, Aerogen Limited, Galway Business Park, H91 HE94 Galway, Ireland

[6] R&D Science & Emerging Technologies, Aerogen Ltd., IDA Business Park, Dangan, Galway, Ireland

[7] School of Pharmacy and Biomolecular Sciences, Royal College of Surgeons, Dublin, Ireland

[8] School of Pharmacy and Pharmaceutical Sciences, Trinity College, Dublin, Ireland

[9] Institut d'Investigacions Biomèdiques de Barcelona, Consejo Superior de Investigaciones Científicas (IIBB-CSIC), 08036 Barcelona, Spain

[10] Servei de Medicina Intensiva, Corporació Sanitària i Universitària Parc Taulí, 08208 Sabadell, Spain

* Correspondence: roig@icmab.es (A.R.)

Nanoparticle tracking analysis

Figure S1. Intensity plot of the hydrodynamic diameter of PLGA/HSA NCs obtained by NTA before and after nebulization at different NC's concentrations (0.1, 1, and 5 mg mL^{-1}) in a) water and b) saline. N=3.

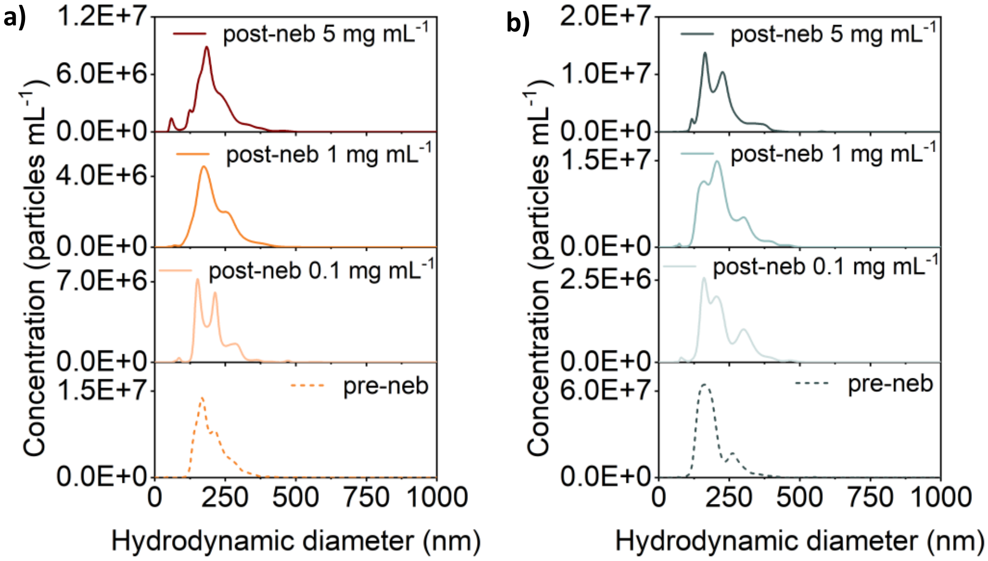

Dynamic and electrophoretic light scattering

The hydrodynamic diameter (d_H) and zeta (ζ) potential of the NCs were also measured by dynamic and electrophoretic light scattering, respectively, using a Zetasizer Nano ZS (Malvern Instruments, Malvern, UK). The samples before and after nebulization were diluted to an NC's concentration of 0.1 mg mL^{-1} using the corresponding medium (ultrapure water or 0.9% saline). The polydispersity index (PdI) was related to the standard deviation (σ) and the mean hydrodynamic diameter (d_H) following Equation S1. Therefore, the standard deviation is an indicator of the homogeneity of the sample.

$$\mathrm{PdI} = \frac{\sigma^2}{\mathrm{d}_\mathrm{H}^2} \qquad \text{(S1)}$$

The hydrodynamic size of the NCs was studied by DLS, and the intensity plots using water and saline are shown in

Figure a and b, respectively. The mean hydrodynamic diameter of PLGA/HSA NCs pre- and post-nebulization in water was between 230 and 260 nm, and it remained very stable regardless of the NCs concentration (

Figure S2 c). In the case of saline medium, the size of the particles experienced a somehow larger variation from 250 nm (pre-nebulization) to 320 nm (post-nebulization). Again, the medium influences the size of the NCs after the nebulization process.

The ζ-potential, indicating the surface charge of the particles, was measured using water to allow a correct performance of the equipment (salts interfere with the measurement due to their conductivity). The ζ-potential of PLGA/HSA NCs remained practically constant for pre- and post-nebulized samples, being (−27 ± 3) mV and (−26 ± 2) mV the corresponding values, respectively.

Figure S2. Hydrodynamic PLGA/HSA NCs diameter obtained by DLS before and after nebulization at different NC's concentrations (0.1, 1, and 5 mg mL^{-1}) in water and saline; a) Intensity plot of the hydrodynamic diameter using water and b) saline; c) comparison of the hydrodynamic diameter mean values. N=3.

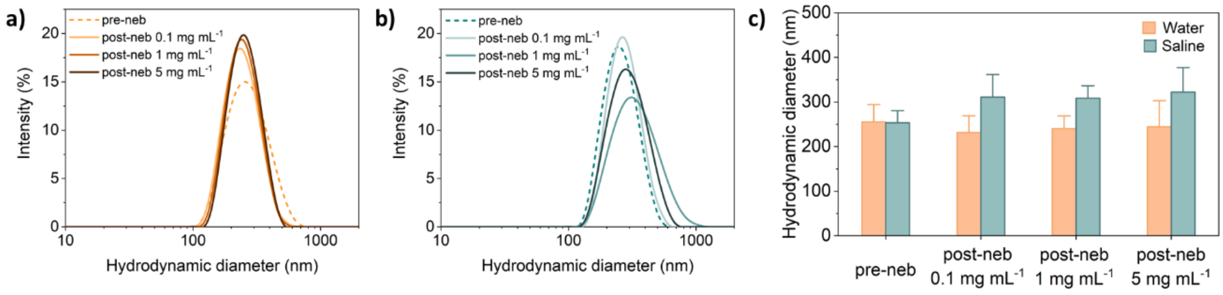

Differences between NTA and DLS hydrodynamic diameters

The differences between the hydrodynamic diameter obtained by NTA and DLS are derived from the principle used to calculate this magnitude. In the case of NTA, a laser beam passes through a chamber where the sample is introduced and the suspended particles scatter the light in such a way that they can be easily viewed through the objective of a 20× magnification microscope, to which a camera is attached. The movement of the particles is captured frame by frame by the microscope's camera. The software simultaneously identifies and tracks the center of each of the observed particles, and determines the average distance traveled by each particle in the xy-plane. This distance allows the particle diffusion coefficient to be determined, from which the hydrodynamic diameter can be calculated using the Stokes-Einstein equation. In addition, the measurement obtained by NTA corresponds to the number-based particle size distribution, which may be different from other techniques, such as DLS or electron microscopy.

In the case of DLS, the particles are illuminated with a laser. Due to Brownian motion, the intensity of the scattered light fluctuates at a rate that depends on the particle size. The analysis of these intensity fluctuations allows the calculation of the correlation function, from which the

diffusion coefficient can be determined. Again, the hydrodynamic diameter can be calculated *via* the Stokes-Einstein equation.

Scanning electron microscopy

The morphology of the PLGA/HSA NCs pre- and post-nebulization in saline at different concentrations was studied by SEM. In Figure S3, sodium chloride crystals are present in the samples due to the evaporation of the solvent. In some cases, NCs tend to accumulate in the edges of these crystals.

Figure S3. SEM images of HSA-loaded PLGA NCs nebulized using saline: a) pre-nebulization; post-nebulization at b) 0.1 mg mL^{-1}, c) 1 mg mL^{-1}, and d) 5 mg mL^{-1}. Scale bar: 2 µm.

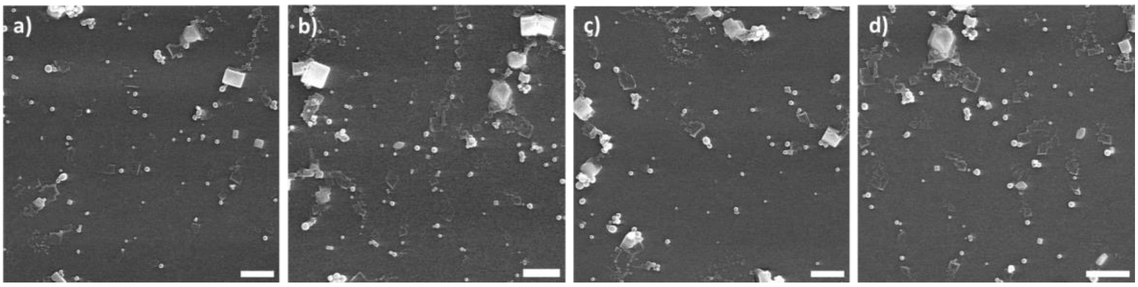

Confocal microscopy

Figure S4. Confocal microscopy images of control animals: Z-stacking analysis of lung (unilobular lung, superior, middle, and inferior lobes) tissue slices of animals nebulized with saline (without NCs). Scale bar: 20 µm. Green: membranes stained with Cell Mask; red: NCs (Cy5); 60× magnification; average zoom = 1×.

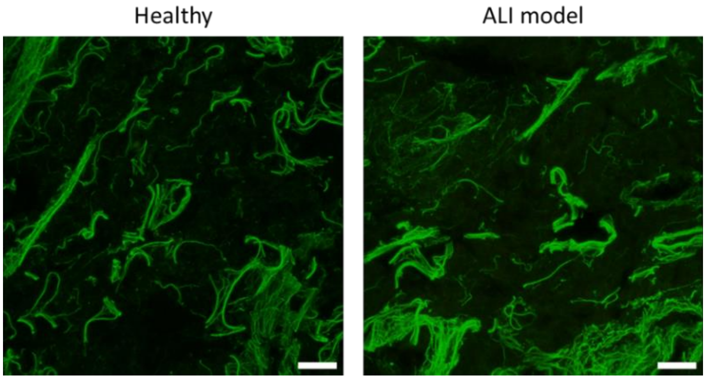

Comparison between aerosols of PLGA NCs and PLGA/HSA NCs

Regarding the effect of HSA on the NC's nebulization, aerosols of both PLGA NCs (empty) and PLGA/HSA NCs (loaded with the protein) were characterized by laser diffraction. The aerosol median diameter, the fine particle fraction, and the flow rate were evaluated. The presence of HSA in the NCs led to:

- Smaller aerosol median diameter for all the concentrations, both in water and saline, although most of the differences were not statistically significant.
- Higher fine particle fraction, both in water and saline, for all the concentrations, although the differences were not statistically significant.
- Smaller flow rate for almost all the samples tested.

Overall, the presence of HSA did not significantly alter the aerosolization process.

Figure S5. Aerosol characterization comparison between PLGA NCs and PLGA/HSA NCs at different concentrations (0.1, 1, and 5 mg mL^{-1}) in water and saline: a) aerosol droplet size (defined as volume median diameter); b) fine particle fraction (defined as the percentage of aerosol with a size below 5 µm); c) flow rate in mL min^{-1}. Data shown as the mean ± SEM (N=3); *p-val < 0.05.

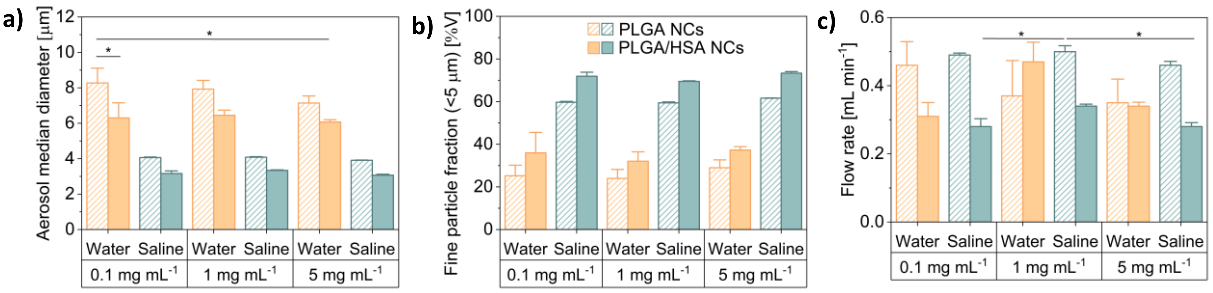

Supplement: Supplementary file 1 — Supplementary Material [file SMSC-4-2400066-s001.pdf]
